# Ten golden rules for optimal antibiotic use in hospital settings: the WARNING call to action

**DOI:** 10.1186/s13017-023-00518-3

**Published:** 2023-10-16

**Authors:** Worldwide Antimicrobial Resistance National/International Network Group (WARNING) Collaborators, Massimo Sartelli, Philip S. Barie, Federico Coccolini, Mohamed Abbas, Lilian M. Abbo, Gulnora K. Abdukhalilova, Yishak Abraham, Salisu Abubakar, Fikri M. Abu-Zidan, Yusuff Adebayo Adebisi, Harissou Adamou, Gulara Afandiyeva, Ervis Agastra, Wadha A. Alfouzan, Majdi N. Al-Hasan, Sajjad Ali, Syed Muhammad Ali, Fatima Allaw, Gbemisola Allwell-Brown, Afreenish Amir, Obed Kwabena Offe Amponsah, Abdelkarim Al Omari, Luca Ansaloni, Shamshul Ansari, Ana Belen Arauz, Goran Augustin, Bih Awazi, Mohammad Azfar, Mamadou Saliou Bailo Bah, Miklosh Bala, Anura S. K. Banagala, Suman Baral, Matteo Bassetti, Luis Bavestrello, Gregory Beilman, Kebebe Bekele, Moussa Benboubker, Bojana Beović, Maria Daniela Bergamasco, Silvia Bertagnolio, Walter L. Biffl, Stijn Blot, Marja A. Boermeester, Robert A. Bonomo, Adrian Brink, Silvio Brusaferro, Jonathan Butemba, Miguel A. Caínzos, Adrian Camacho-Ortiz, Rafael Canton, Antonio Cascio, Alessandro Cassini, Enrique Cástro-Sanchez, Marco Catarci, Rodolfo Catena, Leili Chamani-Tabriz, Sujith J. Chandy, Esmita Charani, William G. Cheadle, Diana Chebet, Ibrahim Chikowe, Francesca Chiara, Vincent Chi-Chung Cheng, Anna Chioti, Maria Elena Cocuz, Raul Coimbra, Francesco Cortese, Yunfeng Cui, Jacek Czepiel, Mira Dasic, Nataliya de Francisco Serpa, Stijn W. de Jonge, Samir Delibegovic, E. Patchen Dellinger, Zaza Demetrashvili, Alessandra De Palma, Danushka De Silva, Belinda De Simone, Jan De Waele, Sameer Dhingra, Jose J. Diaz, Claudia Dima, Natalia Dirani, Cornelius C. Dodoo, Gereltuya Dorj, Therese M. Duane, Christian Eckmann, Beverly Egyir, Mutasim M. Elmangory, Mushira A. Enani, Onder Ergonul, Juan Pablo Escalera-Antezana, Kevin Escandon, Abdul-Wahab Omo-ope Ettu, Joseph O. Fadare, Massimo Fantoni, Mohammad Farahbakhsh, Mario Paulo Faro, Alberto Ferreres, Gianina Flocco, Esteban Foianini, Donald E. Fry, Alberto Federico Garcia, Chiara Gerardi, Wagih Ghannam, Helen Giamarellou, Natalya Glushkova, George Gkiokas, Debra A. Goff, Harumi Gomi, Magnus Gottfredsson, Ewen A. Griffiths, Rosio Isabel Guerra Gronerth, Xavier Guirao, Yogesh K. Gupta, Gregory Halle-Ekane, Sonja Hansen, Mainul Haque, Timothy C. Hardcastle, David T. S. Hayman, Andreas Hecker, Markus Hell, Vanessa P. Ho, Adrien M. Hodonou, Arda Isik, Salequl Islam, Kamal M. F. Itani, Nadia Jaidane, Ib Jammer, David R. Jenkins, Ibrahim Franklyn Kamara, Souha S. Kanj, Desmond Jumbam, Masoud Keikha, Ashish K. Khanna, Sahil Khanna, Gaetanjali Kapoor, Garima Kapoor, Samuel Kariuki, Faryal Khamis, Vladimir Khokha, Reuben Kiggundu, Ronald Kiguba, Hong Bin Kim, Peter K. Kim, Andrew W. Kirkpatrick, Yoram Kluger, Wen-Chien Ko, Kenneth Y. Y. Kok, Vihar Kotecha, Ibrahima Kouma, Bojan Kovacevic, Jehona Krasniqi, Marcela Krutova, Igor Kryvoruchko, Ravina Kullar, Kwaku A. Labi, Francesco M. Labricciosa, Sulaiman Lakoh, Botond Lakatos, Mary Ann D. Lansang, Ramanan Laxminarayan, Young Ran Lee, Marc Leone, Ari Leppaniemi, Gabriel Levy Hara, Andrey Litvin, Varut Lohsiriwat, Gustavo M. Machain, Fawzi Mahomoodally, Ronald V. Maier, Md Anwarul Azim Majumder, Sydney Malama, Justen Manasa, Vikas Manchanda, Ramiro Manzano-Nunez, Luis Martínez-Martínez, Ignacio Martin-Loeches, Sanjay Marwah, Emilio Maseda, Maleda Mathewos, Ryan C. Maves, Deborah McNamara, Ziad Memish, Dominik Mertz, Shyam Kumar Mishra, Philippe Montravers, Maria Luisa Moro, Elias Mossialos, Fabrizio Motta, Steward Mudenda, Patrick Mugabi, Mc Juan Muco Mugisha, Eleftherios Mylonakis, Lena M. Napolitano, Dilip Nathwani, Leontine Nkamba, Emmanuel Fru Nsutebu, Donal B. O’Connor, Sade Ogunsola, Peter Østrup Jensen, Juliana Maria Ordoñez, Carlos A. Ordoñez, Pablo Ottolino, Abdoul-Salam Ouedraogo, José Artur Paiva, Miriam Palmieri, Angelo Pan, Narayan Pant, Arpád Panyko, Ciro Paolillo, Jay Patel, Federico Pea, Patrizio Petrone, Nicola Petrosillo, Tadeja Pintar, Haralds Plaudis, Mauro Podda, Alfredo Ponce-de-Leon, Susan L. Powell, Adrián Puello-Guerrero, Celine Pulcini, Kemal Rasa, Jean-Marc Regimbeau, Jordi Rello, Manuel Renato Retamozo-Palacios, Glendee Reynolds-Campbell, Julival Ribeiro, Jennifer Rickard, Nuno Rocha-Pereira, Victor D. Rosenthal, Gian Maria Rossolini, Godfrey M. Rwegerera, Megan Rwigamba, Michela Sabbatucci, Žilvinas Saladžinskas, Rasha E. Salama, Tondore Sali, Samson Sahile Salile, Ibrahima Sall, Hossein Samadi Kafil, Boris E. Sakakushev, Robert G. Sawyer, Marco Scatizzi, Jeremiah Seni, Edward J. Septimus, Gabriele Sganga, Daniel Mønsted Shabanzadeh, Vishal G. Shelat, Agumas Shibabaw, Francis Somville, Selma Souf, Stefania Stefani, Evelina Tacconelli, Buon Kim Tan, Pierre Tattevin, Carlos Rodriguez-Taveras, João Paulo Telles, Orlando Téllez-Almenares, Jeffrey Tessier, Nguyen Toan Thang, Cristian Timmermann, Jean-François Timsit, Joel Noutakdie Tochie, Matti Tolonen, Gabriel Trueba, Constantinos Tsioutis, Fabio Tumietto, Felipe Francisco Tuon, Jan Ulrych, Selman Uranues, Maarten van Dongen, Harry van Goor, George C. Velmahos, Andras Vereczkei, Bruno Viaggi, Pierluigi Viale, Jordi Vila, Andreas Voss, Jasmina Vraneš, Richard R. Watkins, Nyambura Wanjiru-Korir, Olivia Waworuntu, Agnes Wechsler-Fördös, Klara Yadgarova, Mohammed Yahaya, Ali I. Yahya, Yonghong Xiao, Andee Dzulkarnaen Zakaria, Tanya L. Zakrison, Victor Zamora Mesia, Walter Siquini, Ara Darzi, Leonardo Pagani, Fausto Catena

**Affiliations:** Department of Surgery, Macerata Hospital, Macerata, Italy

**Keywords:** Antibiotic therapy, Antimicrobial resistance, Antimicrobial stewardship programs, Hospital-acquired infections, Infection prevention and control, Systemic antibiotic prophylaxis, Surgical site infections

## Abstract

Antibiotics are recognized widely for their benefits when used appropriately. However, they are often used inappropriately despite the importance of responsible use within good clinical practice. Effective antibiotic treatment is an essential component of universal healthcare, and it is a global responsibility to ensure appropriate use. Currently, pharmaceutical companies have little incentive to develop new antibiotics due to scientific, regulatory, and financial barriers, further emphasizing the importance of appropriate antibiotic use. To address this issue, the Global Alliance for Infections in Surgery established an international multidisciplinary task force of 295 experts from 115 countries with different backgrounds. The task force developed a position statement called WARNING (Worldwide Antimicrobial Resistance National/International Network Group) aimed at raising awareness of antimicrobial resistance and improving antibiotic prescribing practices worldwide. The statement outlined is 10 axioms, or “golden rules,” for the appropriate use of antibiotics that all healthcare workers should consistently adhere in clinical practice.

## Introduction

Antibiotics are essential and life-saving medicines. However, improper use is pervasive. Ensuring appropriate antibiotic prescribing is a fundamental aspect of good clinical practice [1]. Since Sir Alexander Fleming's discovery of penicillin in 1928, antibiotics have revolutionized medicine and been instrumental in saving countless lives [2].

There are also substantial disparities in antibiotic usage worldwide. Whereas some regions face the challenge of excessive antibiotic use, other areas suffer from limited access to essential antibiotics [3]. This concerning disparity creates a gap that jeopardizes the sustainability and safety of global antibiotic supplies, ultimately compromising access to effective treatments and leading to suboptimal prescription practices [4].

Effective antibiotic treatment is an essential component of universal healthcare. There is a global collective responsibility to use antibiotics appropriately to maintain their effectiveness. Pharmaceutical companies have few incentives to develop new antibiotics due to numerous scientific, regulatory, and financial barriers [5–8]. Thus, it is questionable whether industry will replace ineffective antibiotics in time.

Antibiotics are used commonly in acute care hospitals for the treatment of both community- and hospital-acquired infections (HAIs), as well as for surgical prophylaxis [9]. However, when prescribed incorrectly, antibiotics offer little benefit to patients while exposing them to risks of adverse effects [10]. Studies have demonstrated that adverse events are associated with antibiotic therapy in up to 20% of patients receiving systemic treatment [11, 12]. These events, in turn, can prolong hospitalizations, cause additional clinic or emergency department visits and hospital re-admissions, and result in a need for additional hospital services [13] that increase hospital cost [14].

Optimizing inpatient antibiotic prescribing results in improved treatment effectiveness and patient safety, minimizes the risk of antibiotic-associated infections (e.g., *Clostridioides difficile* infection: [CDI]) and the selection and transmission of antimicrobial-resistant bacteria in individual patients within and across hospitals, countries, and globally [15].

We propose that clinical leaders drive antimicrobial stewardship and education programs to help standardize and improve prescribing behaviors. Furthermore, we argue that guidance on the appropriate use of antibiotics from clinical leaders within a specialty is vital to address the global threat of antimicrobial resistance (AMR).

**Fig. 1:**
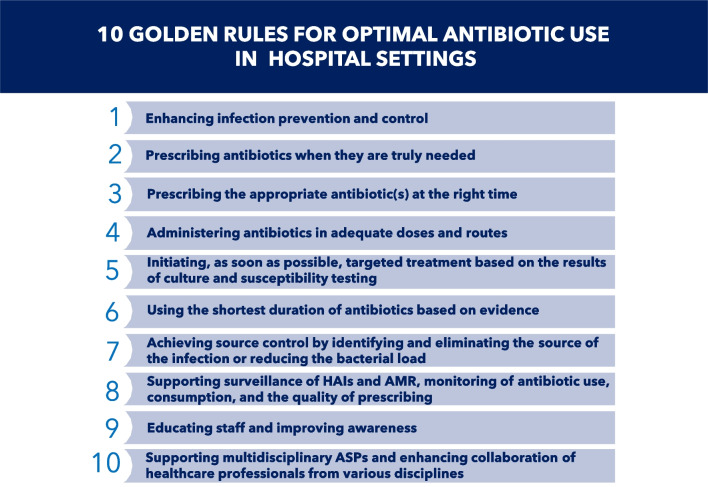
The 10 “golden rules” for optimal antibiotic use in hospital settings, which clinicians should always follow in their clinical practice

We present 10 core principles for the appropriate use of antibiotics, which clinicians should always follow in their clinical practice (Fig. 1).

## Methods

In January 2023, the Global Alliance for Infections in Surgery [16] established an international multidisciplinary task force with the aim of developing a shared vision regarding the need for appropriate antibiotic use in hospital settings to address the threat of AMR in particular antibacterial resistance. Two hundred and ninety-five experts from 115 countries on six continents participated, including specialists in anesthesiology, clinical pharmacology, critical care medicine, emergency medicine, epidemiology, global health, health policy and management, hospital pharmacy, infection prevention and control, infectious diseases, internal medicine, microbiology, nursing, public health, and emergency and general surgery.

**Fig. 2:**
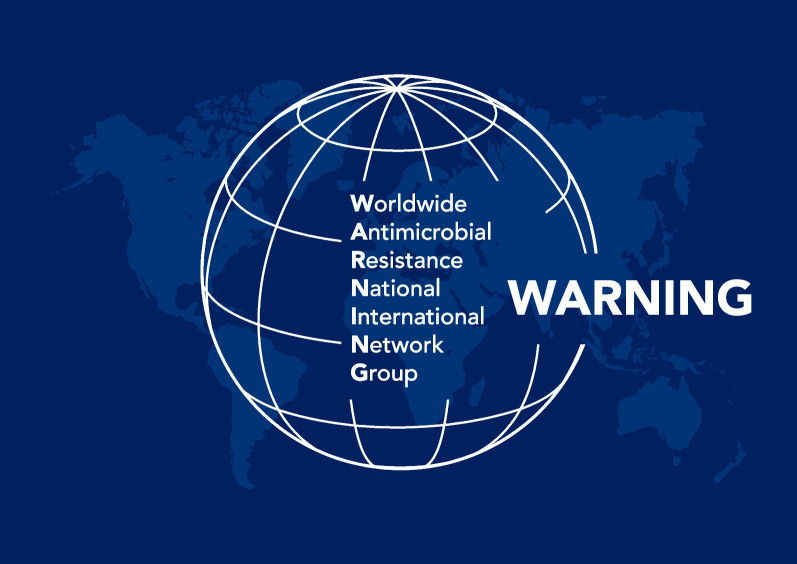
Worldwide Antibiotic Resistance National/International Network Group (WARNING)

Supporting documentation was identified through comprehensive searches conducted using PubMed and Google Scholar. The search identified articles published in English between January 2000 and February 2023. Two experts, who collaborated in drafting the initial manuscript, reviewed the selected articles. Subsequently, the first version was shared with the experts’ group and was revised with the incorporation of additional references. The final document was reviewed thoroughly by each task force member to ensure accuracy, timeliness, and consensus. The project has been named WARNING (Worldwide Antimicrobial Resistance National/International Network Group) (Fig. 2).

By adhering to the 10 core principles described, healthcare professionals in hospital (and community) settings can support responsible and effective antibiotic use, mitigate the risks of adverse effects and AMR, and promote better patient outcomes in their clinical practices. To enhance awareness and promote best practices, we developed impactful iconography that conveys salient messaging, facilitates implementation, and enhances the retention and application of recommended principles and practices.

### The global burden of antimicrobial resistance (AMR)

**Fig. 3:**
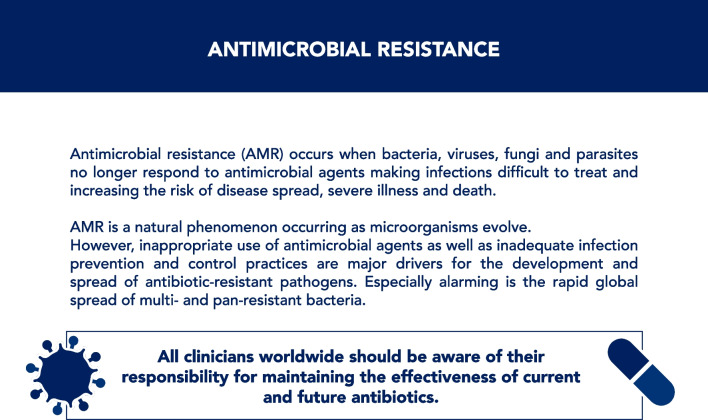
Antimicrobial resistance

AMR occurs as bacteria, viruses, fungi, and parasites evolve antimicrobial defense mechanisms that reduce treatment efficacy and increase the risk of treatment failure, disease progression, severe illness, or death. However, misuse and overuse of antimicrobial agents, combined with ineffective infection prevention and control (IPC) practices, are recognized as major drivers of the increasing prevalence of AMR [1] (Fig. 3).

Although antibiotic-resistant infections are a recognized public health threat, and this call to action addresses the appropriate use of antibacterial agents, less is known about the burden of AMR fungal infections [1]. Fungal infections are increasing in frequency, largely because of the increasing size of the population at risk, which includes persons with cancer, those requiring transplants, persons living with human immunodeficiency virus infection or who are immunosuppressed due to disease or therapy, and critically ill patients. Invasive fungal infections are associated with considerable morbidity and death.

Recently, *Candida auris* has emerged worldwide as a multidrug-resistant (MDR) pathogen [17–19] whose high transmissibility, broad-spectrum clinical manifestations, and potentially high mortality have led the US Centers for Disease Control and Prevention (CDC) to classify it as one of five pathogens in its Urgent Threats category [20]. Data published recently by the CDC highlight that *C. auris* is spreading at an alarming rate [21] since it was first described in 2009 [22] as an invasive infection [23]. Infections due to *C. auris* have increased to the point of higher prevalence than the common fungal pathogen, *C. albicans*, at some centers [24]. *Candida auris* is uniquely challenging due to five factors: high transmissibility leading to widespread outbreaks in numerous hospitals worldwide [25, 26]; a broad spectrum of clinical manifestations associated with a mortality rate as high as 70% [26, 27]; environmental hardiness, including persistence for weeks on dry surfaces [28, 29]; difficulty identifying *C. auris* by microbiology laboratories [29]; and a high rate of MDR and therapeutic failure [28, 30–32]. The environmental fitness of *C. auris* is associated with biofilm formation and production of proteinases and phospholipases [27, 33], in addition to environmental stress resistance and antifungal drug resistance.

**Fig. 4:**
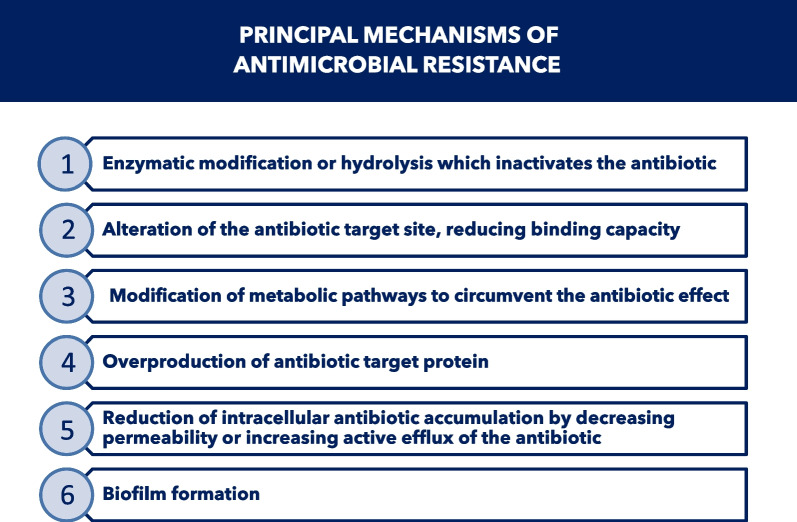
Principal mechanisms of antimicrobial resistance

Bacteria may be intrinsically resistant to one or more classes of antibiotics or may acquire such resistance. Bacteria have developed different resistance mechanisms to avoid antibiotic action (Fig. 4). In addition to intrinsic resistance mechanisms, bacterial pathogens can acquire resistance to antibiotics through either mutation of existing genes [34], or by acquiring new genes from other strains or species through horizontal gene transfer [34].

“Heteroresistance” describes the presence of subpopulations of bacterial cells with higher levels of antibiotic resistance than those of the rest of the population in the same culture [35]. Recent work indicates that heteroresistance is very common for several different bacterial species and antibiotic classes. The resistance phenotype is often unstable, and in the absence of antibiotic pressure, it can rapidly revert to susceptibility [36]. Heteroresistance occurs in both Gram-positive and Gram-negative bacteria. Its clinical relevance may be considerable, since more resistant subpopulations may be selected during antibiotic therapy. However, the use of nonstandard methods to define heteroresistance, which are costly and involve considerable labor and resources, precludes evaluating the clinical magnitude and severity of this phenomenon [35]. Since heteroresistance may have serious implications in antibiotic therapy, the development of standardized criteria and protocols for detecting and measuring heteroresistance is essential.

Infections caused by AMR bacteria pose a global challenge [37]. In 2008, the “ESKAPE” acronym was coined to name those bacteria that may “escape” the effects of antibiotics including *Enterococcus faecium, Staphylococcus aureus, Klebsiella pneumoniae, Acinetobacter baumannii-calcoaceticus* complex*, Pseudomonas aeruginosa,* and *Enterobacter* spp. [38]. The list of AMR bacteria is no longer up-to-date, as *Escherichia coli, Mycobacterium tuberculosis* and *Neisseria gonorrhoeae* are currently among the most prevalent bacterial pathogens affected by AMR issues.

**Fig. 5:**
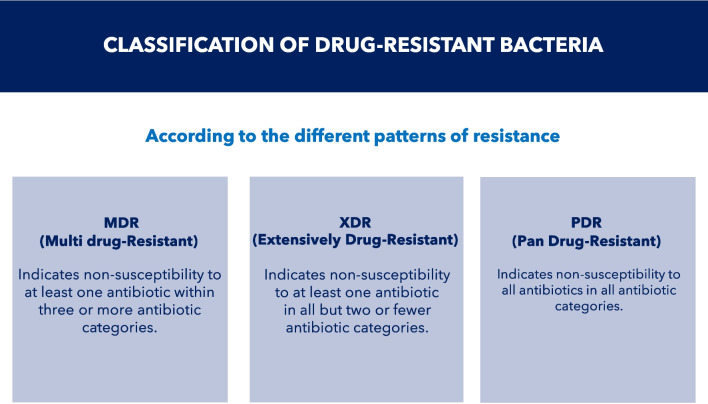
Classification of drug-resistant bacteria

In 2012, the European Centre for Disease Prevention and Control (ECDC) and the CDC developed standardized nomenclature to describe acquired resistance profiles in bacteria [39]. MDR was defined as acquired non-susceptibility to at least one antibiotic in three or more antibiotic classes (e.g., cephalosporins, fluoroquinolones, tetracyclines). Extensively drug-resistant (XDR) bacteria were defined as non-susceptibility to at least one antibiotic in all but two or fewer antibiotic classes (bacterial isolates remain susceptible to only one or two classes). Pandrug-resistant (PDR) bacteria were defined as non-susceptibility to all antibiotics in all antibiotic classes (Fig. 5). These classifications provide a standardized nomenclature for categorizing and communicating resistance patterns of bacteria, aiding in surveillance, research, and the development of appropriate tactics to combat AMR [40]. Kadri et al. [41] proposed a new category of Gram-negative bacteremia, that is difficult-to-treat, based on non-susceptibility to “first-line” antibiotics, generally beta-lactams or fluoroquinolones, that necessitates the use of second-line, often more toxic, agents.

**Fig. 6:**
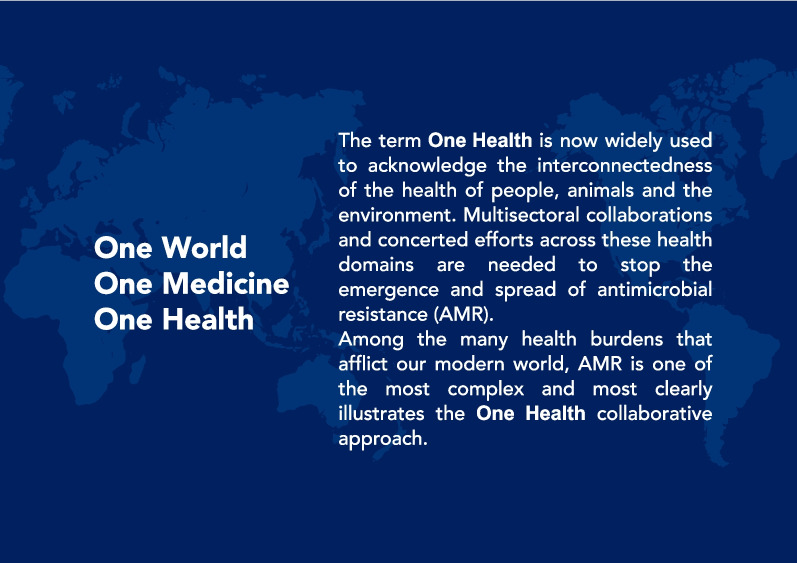
One Health

AMR is a complex and multifaceted issue that involves not only humans, but also animals and the environment [42, 43]. On March 17, 2022, four international agencies, the Food and Agriculture Organization of the United Nations (FAO), the World Organization for Animal Health (WOAH), the UN Environment Programme (UNEP), and the World Health Organization (WHO), signed a groundbreaking agreement to strengthen cooperation and promote sustainable practices that balance and optimize the health of humans, animals, plants and the environment. The concept of "One Health" recognizes the interconnectedness of the health of people, domestic animals, and the environment [44]. Multisectoral collaborations and concerted global efforts across multiple health domains are needed to tackle AMR [45–48] (Fig. 6).

In 2015, the WHO published the Global Action Plan on Antimicrobial Resistance [49]. Its five goals include: Improving awareness and understanding of AMR through effective communication, education, and training; strengthening knowledge through surveillance and research; preventing infection through effective sanitation, hygiene, and IPC measures; optimizing the use of antibiotics in human and animal health; and increasing investment in new drugs, diagnostic tools, vaccines, and other interventions. Member states committed to develop national action plans (NAPs) on AMR, which should be comprehensive, funded, and implemented with monitoring so that lessons learned can reshape priorities. Inter-country variability in economic and political resilience, and resource constraints, constitute a considerable barrier to implementation of the NAPs [50, 51]. Despite the strong commitment to addressing AMR, endorsement and implementation of NAPs have also been impeded by the prioritization of issues related to the coronavirus disease-2019 (COVID-19) pandemic [52].

There is increasing evidence that the pandemic accelerated the emergence and spread of AMR at least in hospital settings [53] particularly *Acientobacter* spp. Langford et al. reported that more than 60% of patients with COVID-19 who had a bacterial infection harbored a highly resistant organism [54]. However, multiple limitations intrinsic to the interpretation of COVID-19 data prevent accurate quantification of its impact on the global epidemiology of AMR [55, 56].

Alarming levels of AMR have been reported in all countries, regardless of their average income level [57]. The 2019 pre-pandemic analysis, published in 2022 by Murray et al. [57], revealed AMR as a leading cause of death worldwide (204 countries and territories) with 4.95 million estimated deaths associated with bacterial AMR, including 1.27 million deaths attributable directly to bacterial AMR. Among the 23 bacteria studied, six (*E. coli, S. aureus, K. pneumoniae, S. pneumoniae, A. baumannii,* and *P. aeruginosa*) were found to be responsible for 929,000 deaths due to AMR and 3.57 million deaths total. Notably, methicillin-resistant *S. aureus* (MRSA) alone caused more than 100,000 deaths in 2019. AMR bacterial infections were associated with the highest infection-related mortality rates in sub-Saharan Africa, with 99 deaths/100,000 people. By comparison, in high-income countries, AMR was associated with 56 deaths/100,000 individuals. However, Murray et al. may have underestimated the true burden of AMR [58]. Modern medical therapies, including trauma care, oncologic surgical interventions and chemotherapy, organ transplantation, and other invasive procedures, require effective antibiotics to prevent and treat infection. Untreatable infections reduce the value of these medical interventions by impacting efficacy adversely, although this is difficult to quantify [58].

The true burden of AMR in low- and middle-income countries (LMICs) would remain unknown unless surveillance is resourced adequately [59]. In particular, bacterial identification and susceptibility testing are not performed routinely in LMICs, owing to a lack of personnel, equipment, and supplies; moreover, testing may represent an out-of-pocket expense for patients in some healthcare systems [60]. As a result, antibiotic therapy is mostly empiric and broad-spectrum antibiotics may be misdirected. The resultant suboptimal care of infections can lead to clinical failure, higher mortality, and increased AMR. Some progress has been made in LMICs over the last decade regarding data collection to inform AMR, and monitoring of antibiotic use. However, more must be done.

The COVID-19 pandemic has demonstrated that morbidity and mortality from infectious diseases disproportionately impact upon certain populations [61]. The measures recommended to control the spread of Severe Acute Respiratory Syndrome Coronavirus 2 (SARS-CoV-2), including social distancing and frequent hand washing, pose challenges for those living in densely populated communities with inadequate housing, poor sanitation, and limited access to clean water. The poorest people are particularly vulnerable to the threat of AMR, as poverty increases the risk of contracting infectious diseases and being exposed to antibiotics. A 2018 systematic review by Alividza et al. [62] highlighted the complex relationship between AMR and various dimensions of poverty, including education level, income, and housing and water quality. Addressing these disparities will be crucial for reducing the burden of AMR and improving public health outcomes in vulnerable communities.

An important report from India (the ‘Chennai Declaration’) was published in November 2012, representing a major national step forward as a landmark commitment to antibiotic stewardship, with international importance and global implications [63]. Although there has been a national antibiotic policy in India since 2011, the recommendations were difficult to implement owing to a lack of a clear plan of action. The lack of impact of such a well-intentioned but difficult-to-implement policy gave rise in August 2012 to a meeting of Indian medical societies and national authorities to develop a ‘roadmap’ outlining the urgent actions required. The final declaration was released in November 2012. The effort represents an extraordinary example of national consensus and commitment that recognizes the clinical and public health issues of AMR.

Recognizing the gravity of AMR, the United Kingdom commissioned in 2014 a comprehensive analysis of this global problem [64]. The stunning finding of this report was that, provided no action was taken, AMR would result in as many as 10 million deaths by 2050. Separately, the World Bank warned that "*in the high AMR-impact scenario, an additional 24 million people could be pushed into extreme poverty by 2030*" [65]. Although there is undoubtedly a large clinical and public health burden associated with AMR, it is challenging to quantify the associated excess morbidity and mortality. Detailed, reliable data, preferably based on comprehensive, population-based surveillance from LMICs and high-income countries [66] will be needed to enhance AMR control measures.

In 2022, the Group of Seven (G7; Canada, France, Germany, Italy, Japan, UK, USA) issued the G7 Health Ministers’ Declaration [67]. Their communiqué covered a range of topics but focused on four priority areas: (1) overcoming COVID-19; (2) future pandemic preparedness; (3) AMR; (4) and health risks from climate change [67]. In a subsequent communiqué, the G7 health ministers called AMR an "*urgent public health and socio-economic problem*" that may have global effects but could have a greater impact on LMICs. Acknowledging AMR as a shared responsibility, they committed together to "*taking further urgent and tangible action*" to address the issue. Among the actions, they pledged to establish new or improved national integrated surveillance systems on AMR and antibiotic use in human beings, animal husbandry, farming, and environmental sectors; promotion of appropriate antimicrobial use through stewardship; strengthening implementation of IPC programs across the One Health spectrum; and strengthening the research and development pipeline for new antibiotics. This approach aims to achieve optimal health outcomes for people, animals, and the environment, while considering the diverse socioeconomic, political, and cultural contexts affecting AMR [68], including limited technical expertise, insufficient clinical and research laboratory infrastructure, other financial constraints, and necessary political commitment [69]. As effective antibiotics are a global public good on the verge of scarcity, AMR is rightly considered a serious threat [70]. Preserving antibiotics is a collective responsibility [8, 71].

On 13 June 2023, the European Council adopted a resolution calling for stronger EU action to combat AMR in human and animal health and the environment, employing a 'One Health' approach to AMR. The resolution encourages the prudent use of antimicrobial agents in human and animal health through a series of voluntary measures, with the aim of reducing AMR [72].

### Antibiotic use in the hospital and community setting

Healthcare workers (HCWs) play a crucial role in combatting AMR [1]. Unfortunately, antibiotics are often prescribed inappropriately in human and animal health settings [73]. When prescribing antibiotics, understanding the differences among prophylactic, empiric, and targeted therapy can help ensure appropriate use and help prevent the development of AMR. *Antibiotic prophylaxis* refers to the antibiotic administration to patients without signs of infection, in order to prevent its occurrence. *Empiric antibiotic therapy* is prescribed to treat known or suspected infections based on the patient's symptoms and likely causative pathogens before definitive diagnostic test results, including antibiotic susceptibility testing, are available. *Targeted antibiotic therapy* is initiated based on microbial identification and susceptibility test results to identify the specific pathogen and ensure that the most effective (ideally, also the most cost-effective), least toxic, and narrowest spectrum antibiotic is used as therapy. Optimal targeted therapy requires early identification and characterization of bacteria. However, despite advancements in rapid microbial diagnostics, the turnaround time for microbiologic testing and reporting can still take up to 72 h, if it is available at all. As a result, clinicians often initiate empiric antibiotic therapies that can have negative consequences for patients' health and exacerbate the risk of AMR [74].

Although antibiotic decision-making is reported to be driven by different determinants in medical versus surgical settings [75], hospital antibiotic prescribing practices are often inadequate worldwide [76]. A point-prevalence survey of 33 hospitals in five Latin American countries (Cuba, El Salvador, Mexico, Paraguay and Peru) documented adherence to prescribing guidelines in 68.6% of cases. Third-generation cephalosporins were the most frequently prescribed antibiotic class (26.8%), followed by carbapenems (10.3%) and fluoroquinolones (8%). Targeted therapy was achieved in only 17.3% of cases [77].

All clinicians must strive for improvement by incorporating antibiotic stewardship principles into daily practice [78]. Antimicrobial stewardship programs (ASPs) [79–81] should be integrated into all hospitals’ quality improvement programs worldwide. ASPs promote responsible antibiotic use by improving the diagnostic decision-making process (now called *diagnostic stewardship*); emphasizing the importance of prescribing antibiotics only when needed; to the right patient and clinical situation, at the right time, in the right dose and interval, and for the correct duration [82–84]. ASPs also play a vital role in increasing awareness of HCWs and community members regarding AMR [85, 86]. Diagnostic stewardship is an integral part of ASPs and emphasizes the importance of selecting the right diagnostic tests for the right patient at the right time [87], encouraging the use of rapid molecular diagnostics to initiate targeted antibiotic therapy as soon as possible while avoiding excessive use of broad-spectrum antibiotics when not (or no longer) needed. However, equally important is accurate interpretation of test results to prevent overdiagnosis and unnecessary cost [88], and improving the diagnostic decision-making process overall, integrating all needed information (clinical, biologic, imaging).

Although 15 years have passed since the CDC, the Society for Healthcare Epidemiology of America (SHEA), and the Infectious Diseases Society of America (IDSA) published joint guidelines for the development of institutional ASPs, best practices for ASPs are still being defined and are likely to vary based on local practice patterns, policy, and available resources [89]. The preferred means of improving antimicrobial stewardship include a comprehensive program that incorporates collaboration among specialists and support staff within an institution. In this context, the direct involvement of all prescribers in ASPs can be highly impactful [90].

Thus, we present the following 10 principles for the appropriate use of antibiotics. These principles should be adhered to by all HCWs in their clinical practices, and they should be considered as core components of activity within ASPs.

#### Enhancing infection prevention and control (IPC)

**Fig. 7:**
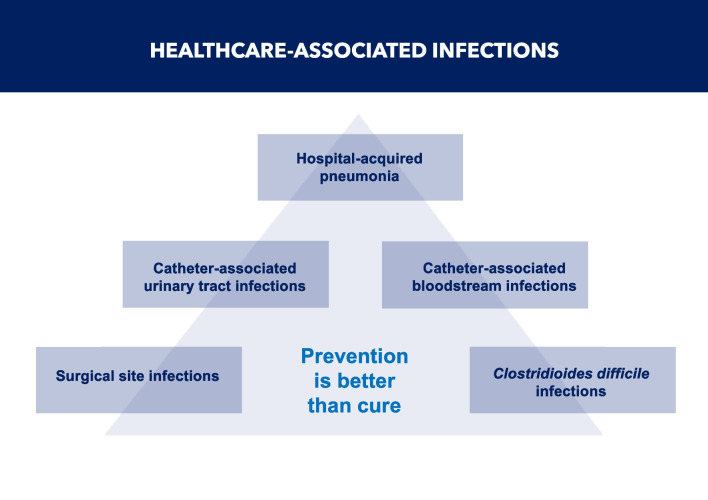
Healthcare-associated infections

It is crucial for all HCWs to adhere to evidence-based measures of IPC to prevent the occurrence of HAIs. Effective IPC education and training significantly reduce HAIs [91–94], the most common of which are surgical site infections (SSIs), catheter-associated urinary tract infections, central line-associated blood stream infections, hospital- and ventilator-associated pneumonia (VAP), and CDI [95] (Fig. 7).

Patients with HAIs require additional diagnostic and therapeutic procedures, have prolonged hospital stays, incur additional costs, and may have high morbidity and mortality. Moreover, many HAIs are caused by MDR bacteria [96, 97]. In the contexts of quality care and mitigation of AMR, preventing HAIs becomes increasingly important. These infections are associated with worse outcomes and often require broad-spectrum antibiotics [98]. According to the ECDC, the burden of the six major types of HAIs in the European Union/European Economic Area, expressed in disability-adjusted life years, was higher than the combined burden of all 32 other communicable diseases surveilled by the ECDC based on data from 2011 to 2012 [99].

**Fig. 8:**
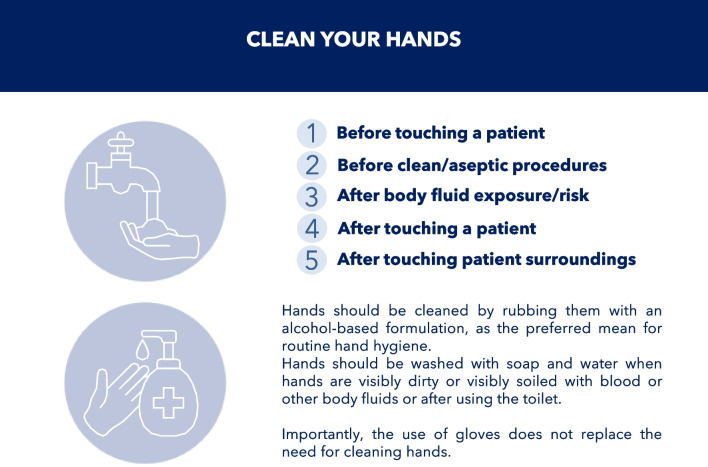
The five moments for hand hygiene

Many HAIs are preventable. A reduction in HAI rates of 35–55% has been documented by implementing multimodal prevention and developing a safety-oriented approach, regardless of the countries’ income levels [100]. Despite this, HCWs adhere poorly to evidence-based IPC measures [95]. A prominent example is hand hygiene, considered an indicator of patient safety and quality of care and the cornerstone of IPC in all healthcare settings. Numerous organizations, including WHO [101] and CDC [102], have published guidelines providing HCWs with specific recommendations to improve hand hygiene practices. Recently, SHEA, IDSA, and the Association for Professionals in Infection Control and Epidemiology (APIC) published practice recommendations for the prevention of HAIs through hand hygiene [103]. The *Five Moments for Hand Hygiene* were promulgated by WHO to encourage HCW adherence to hand hygiene recommendations and minimize the risk of infection and transmission [104] (Fig. 8).

Although hand hygiene is accepted universally as a cost-effective IPC measure, compliance remains unacceptably low. In a systematic review [105], reported compliance was ~ 40% (compared with the WHO benchmark rate of > 80%) and was variable across hospital units/wards and HCWs, calling for multifaceted mitigation activities to foster concordance. All HCWs involved in direct or indirect patient care should recognize the importance of hand hygiene and the need to perform it without fail. Hand hygiene at the point of care is recognized as a best practice for promoting compliance at the moments when hand hygiene is most crucial. According to current best practice, hand hygiene products should be available at the point of care. This requires that a hand hygiene product be easily accessible and as close as possible—ideally within arm’s reach of where patient care or treatment is taking place [106].

SSIs remain the most common HAIs among surgical patients. They represent a major clinical problem in terms of morbidity, mortality, length of hospital stay, and overall direct and indirect costs worldwide. It is obviously important to improve patient safety by acting before, during, and after surgery to reduce the occurrence of SSIs [107–109].

**Fig. 9:**
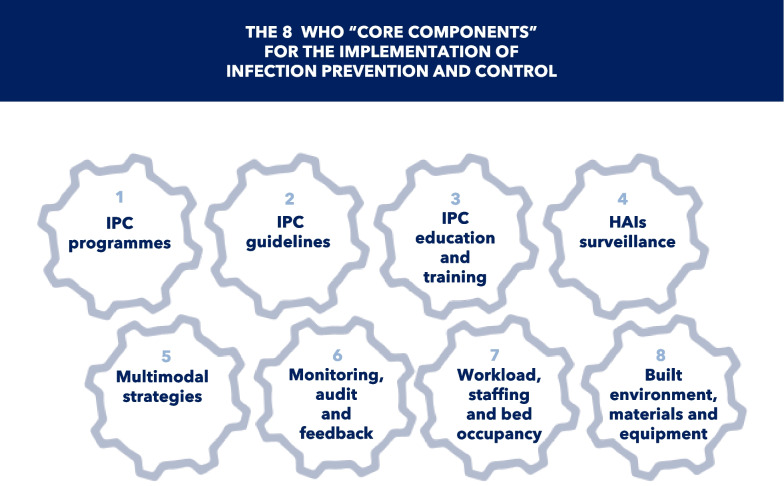
The 8 WHO “core components” for the implementation of infection prevention and control

In 2016, WHO published evidence-based guidelines on the core components of effective IPC programs, to be implemented both at the national and hospital levels [110, 111]. IPC measures were summarized in eight “core components” (Fig. 9). Since the landmark Study on the Efficacy of Nosocomial Infection Control (SENIC) program in the 1970s [112] revealed the effectiveness of an IPC program in reducing HAIs, a dedicated IPC program is considered of paramount importance in every hospital. It should be led by experts in IPC, in close collaboration with HCWs in all relevant areas [113].

Results of a WHO global survey designed to assess implementation of these programs in healthcare facilities worldwide have been published. This survey provides an important overview of IPC program implementation in 4440 healthcare facilities (81 countries) across all six WHO regions and income levels. The findings identify strengths, gaps in IPC implementation, and key opportunities for improvement to inform ongoing global IPC improvement efforts, particularly in LMICs, which showed significantly lower IPC implementation [114].

With the common goal of reducing AMR, IPC programs and ASPs should be partners in reducing HAIs. Support from institutional leadership is crucial for the success of each program and both together, including an effective microbiology laboratory (capacity, funding, and infrastructure) to enable rapid diagnosis, effective communication tools, and appropriate use of technology, including electronic health records. IPC and ASP programs are based on similar models of interdisciplinary work and activities such as education, monitoring, and feedback. Integrating these interventions may reduce redundancy and align forces for maximal influence on HCWs. ASPs, when implemented in concert with IPC interventions in hospitals, particularly hand hygiene, are significantly more effective in reducing the development and spread of MDR bacteria than ASPs alone [115].

Containing the spread of antibiotic-resistant bacteria is challenging because of their propensity for human-to-human transmission [116]. Carbapenem-resistant *Enterobacterales* (CRE),* A. baumannii* (CRAB), and *P. aeruginosa* (CRPA) are among the most difficult-to-treat bacteria due to a high prevalence of AMR. In 2017, the WHO published guidelines for the prevention and control of these bacteria in acute healthcare facilities [117]. The supporting systematic literature review was published in 2019 [118]. The most frequent interventions reported were contact precautions (90%); active surveillance cultures (80%); monitoring, audit, and feedback of measures (80%); patient isolation or cohorting (70%); hand hygiene (50%); and environmental cleaning (40%).

Vaccination deserves mention as one of the most impactful and cost-effective prevention measures. Vaccines are mostly used prophylactically, including post-exposure prophylaxis, to decrease the number of infectious disease cases, and thus antibiotic use and the propagation of AMR. Vaccines are also being developed against resistant bacterial pathogens that cause a substantial disease burden [119] such as MRSA and *P. aeruginosa* [120, 121]. Vaccines are commonly considered to impact AMR, either directly by preventing infection, thereby reducing the prevalence of the resistant pathogen and also antibiotic use, or indirectly by preventing non-bacterial primary infections (e.g., viral), which are often treated incorrectly with antibiotics [119]. *Haemophilus influenzae* serotype B (HiB), influenza, and pneumococcal conjugate vaccines are examples demonstrating the effectiveness of vaccines in reducing antibiotic use and reducing AMR [122–124]. Specific to surgery, vaccination against *S. pneumoniae* (which is increasingly resistant to penicillin), HiB, and *Neisseria meningitidis* following splenectomy is effective in preventing overwhelming post-splenectomy infection that is usually caused by encapsulated organisms [125]. Moreover, immunization against measles prevents measles virus infection, which infection reduces preexisting antibodies offering protection from other pathogens [126]. New vaccines are under development and evaluation, offering possibilities to address life-threatening diseases and help further curb antibiotic use and mitigate AMR [127].

#### Prescribing antibiotics when they are truly needed

**Fig. 10:**
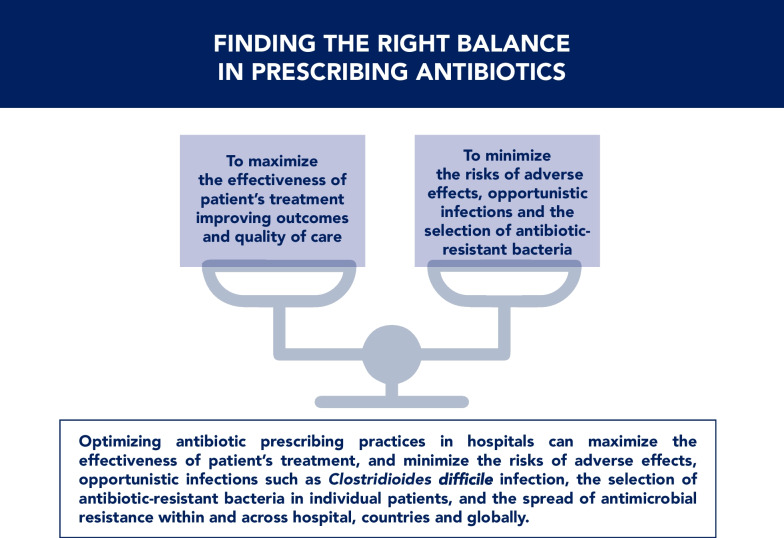
Finding the right balance in prescribing antibiotics

Clinicians prescribing antibiotics are faced with conflicting priorities. On the one hand, they must provide patients with the best possible treatment. On the other hand, they must preserve the efficacy of antibiotics, minimize opportunistic infections such as CDI, reduce the selection of resistant pathogens in individual patients, and prevent the continued global increase of AMR. These conflicts should be evaluated and balanced before prescribing antibiotics [34] (Fig. 10).

The intestinal microbiota has an important role in human health and can protect the patient against colonizing enteric bacteria [128], a phenomenon known as *colonization resistance*. The indigenous bacteria of the microbiome provide an important host defense mechanism by inhibiting colonization by potentially pathogenic bacteria. However, in certain circumstances, a patient’s microbiota can be compromised, no longer protecting against colonization by opportunists.

**Fig. 11:**
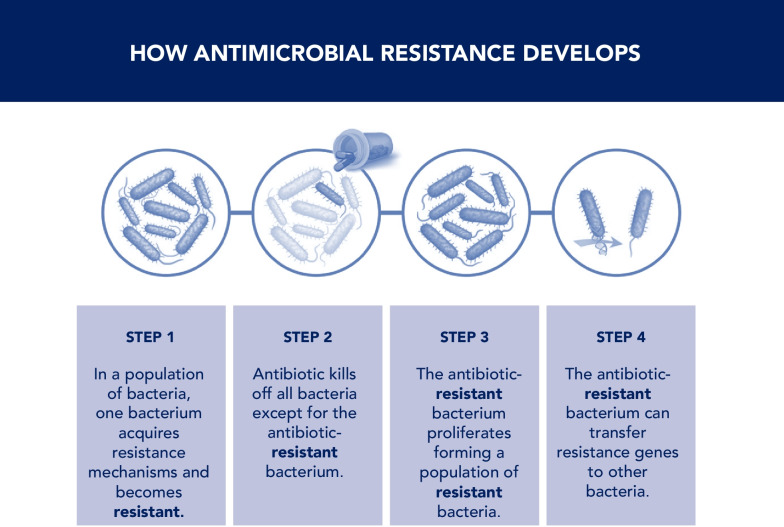
How antimicrobial resistance develops

Antibiotics exert selection pressure on the human microbiome, predisposing to AMR. Antibiotic use can have unintended consequences on commensal intestinal microbiota. Whereas susceptible bacteria are destroyed, the resultant ecologic vacuum promotes the overgrowth of pathogenic bacteria that may already be antibiotic-resistant [129, 130]. Moreover, antibiotics facilitate the transmission of resistance genes conferring resistance to other bacteria [131, 132] (Fig. 11), thereby increasing the risks of cross-transmission between patients [133, 134] and outbreaks of infections caused by MDR bacteria.

Many studies have evaluated the long‐term effect on gut microbiota following a course of antibiotics [135–137]. To demonstrate the long-term effects of antibiotics on the healthy microbiome, the effects of amoxicillin (500 mg) thrice daily for 5 days [138], ciprofloxacin (500 mg) twice daily for 5 days [139], and (second-generation cephalosporin) cefprozil (500 mg) twice daily for 7 days [140] were evaluated in healthy individuals. Changes in the microbiota persisted for up to 12 weeks after the end of the treatment, characterized by the incomplete restoration of microbial equilibrium and the emergence of MDR strains. Moreover, compared with parenteral antibiotics, oral agents result in higher concentrations of antibiotics in the intestine and larger numbers of MDR bacteria in the intestinal microbiota [141]. A study of ciprofloxacin (500 mg twice daily for 10 days) or clindamycin (150 mg four times daily for 10 days) on the fecal microbiota of healthy human beings for 1 year showed a profound impact on the diversity of the microbiome [141]. Changes in microbial equilibrium were most pronounced in the first month after treatment, but persisted until month 20.

The commensal intestinal microbiota plays a pivotal role in protection against CDI [142]. *Candida difficile* is rarely present in the gut of healthy adults (~ 3%) [143]. The correlation between antibiotic exposure and CDI has been demonstrated [144]. Disruption of normal intestinal flora consequential to antibiotic use provides an opportunity for *C. difficile* to proliferate 'and produce toxins [145]. Animal and clinical studies have shown that normal intestinal microbiota inhibits the expansion and persistence of *C. difficile* [146]. These alterations can be evident during administration and for several days after the discontinuation of an antibiotic [147], depending on the administered antibiotic and the person’s microbiota. The risk of CDI is estimated to increase up to sixfold during and in the subsequent month after antibacterial therapy [34]. Although most antibiotics have been associated with CDI, clindamycin, amoxicillin-clavulanic acid, third- and fourth-generation cephalosporins, fluoroquinolones, and carbapenems pose the greatest risk [34].

Surgical antibiotic prophylaxis (SAP) is a crucial component of perioperative infection prevention [148, 149], particularly in clean-contaminated and contaminated surgical procedures with a high infection risk. SAP may also be indicated in certain clean procedures where SSI, even if unlikely, may have devastating consequences, such as procedures with prosthetic implants. Patients with medical conditions associated with a higher risk of SSI, including immunocompromised individuals (e.g., neutropenia), patients with American Society of Anesthesiologists (ASA) score ≥ 3, and obese patients. Despite SAP not being required before all surgical procedures, over-administration is frequent, contributing substantially to overall antibiotic consumption in surgical services. Elective laparoscopic cholecystectomy carries a low risk of SSI. Use of prophylactic antibiotics is not justified in patients undergoing elective, uncomplicated laparoscopic cholecystectomy. The role of SAP in patients undergoing open-groin herniorrhaphy or hernioplasty remains uncertain owing to conflicting results of generally low evidence quality [150–155]. International guidelines [156] recommend SAP in open-groin mesh repair in any patient in a high-risk environment.

Antibiotic therapy should be prescribed after a bacterial infection has been confirmed. Colonization by potential pathogens without associated signs of infection occurs frequently in certain patients (e.g., those with indwelling urinary catheters, endotracheal tubes for mechanical ventilation, chronic wounds). Appropriate evaluation requires obtaining a culture from these sites only when indicated, without contamination by the collection protocol itself (superficial swab cultures and cultures of drains [157] and sinus tracts are inappropriate), and avoiding antibiotic treatment of a “positive” culture result without symptoms and signs of active infection [158]. Asymptomatic bacteriuria is a common scenario for which antibiotics are not recommended, yet it is often treated regardless. Patients with a urinary drainage catheter may have “positive” urine culture results owing to inevitable biofilm formation on the device. Numerous studies show that antibiotic treatment of patients with asymptomatic bacteriuria is not indicated except in specific circumstances, such as pregnancy or transurethral instrumentation, because it can increase the likelihood of subsequent urinary tract infections that can become resistant to common antibiotics [159, 160].

The use of antibiotics in the treatment of mild uncomplicated diverticulitis has been common, but is now being questioned. Mounting evidence suggests that mild uncomplicated diverticulitis is more likely to be an inflammatory rather than an infectious condition, questioning the appropriateness of antibiotic use [161]. Three randomized trials each showed that antibiotic treatment neither prevents complications or recurrences nor reduces symptoms or length of hospital stay [161–163], as did two prospective cohort studies [164, 165]. The results of these studies have led some experts to advocate against the routine use of antibiotics [166, 167].

#### Prescribing the appropriate antibiotic(s) at the right time

**Fig. 12:**
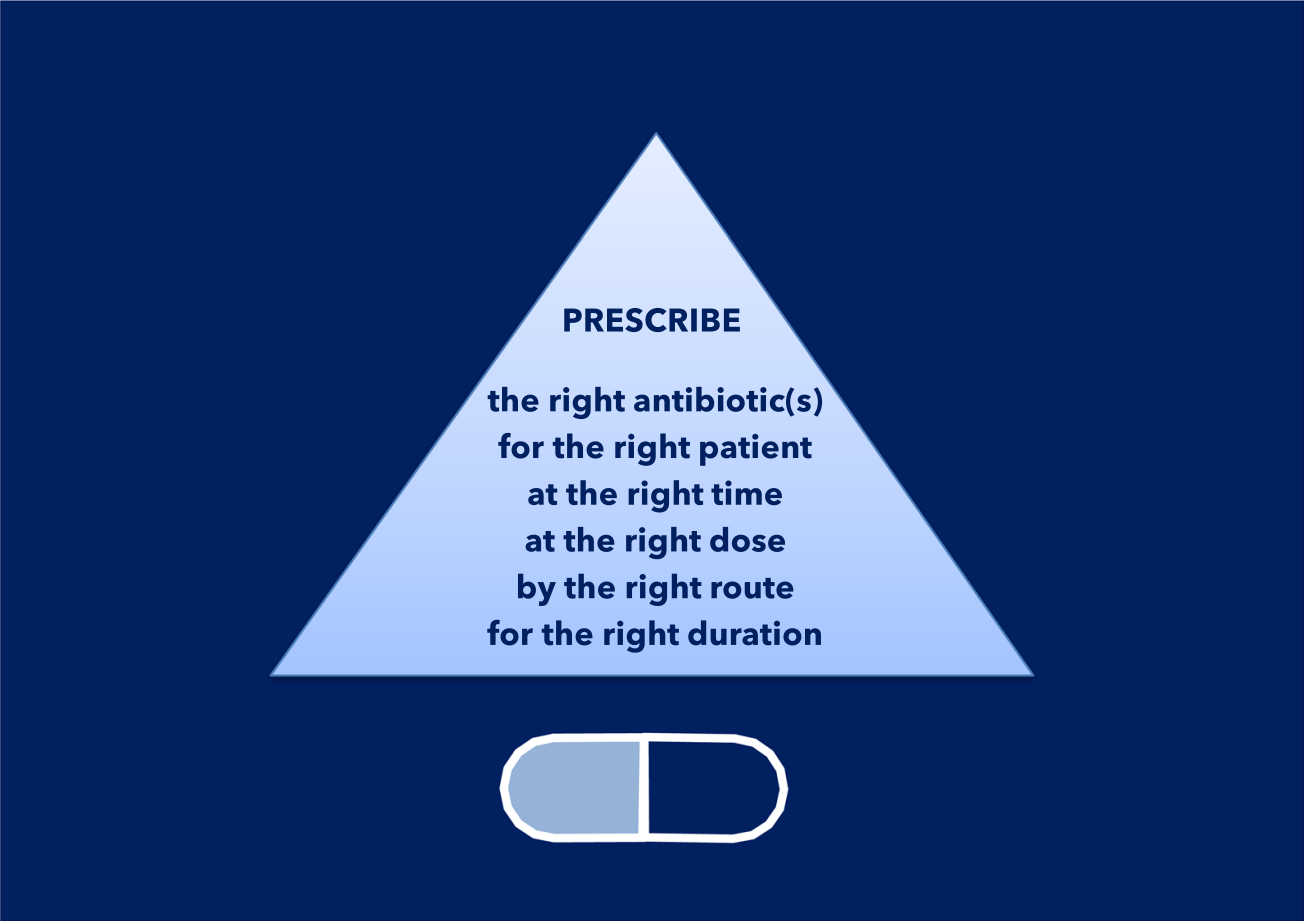
Selecting the most appropriate antibiotic(s) for a specific patient

Once the treatment decision has been made, it is crucial to select the most appropriate antibiotic(s) for that specific patient (Fig. 12). The antibiotic selected for SAP should be active against the common bacteria causing SSIs in the specific procedure. SSIs in clean procedures are usually due to skin flora, including *S. aureus* or coagulase-negative staphylococci. Clean-contaminated and contaminated procedures can involve other bacteria, such as *E. coli*, other Enterobacterales, or anaerobes, depending on the flora of the mucous membranes incised.

The most common antibiotics used for SAP have been first- and second-generation cephalosporins (e.g., cefazolin, cefuroxime) [148, 149]. Cefazolin is the drug of choice for SAP before most procedures. It has proven efficacy, a suitable duration of action, activity against the bacteria commonly encountered in SSI, a reasonable safety profile, and low cost. Routine use of vancomycin in SAP is not recommended. Vancomycin may be considered for SAP in patients with known MRSA colonization or those at high risk therefor, such as in institutions with a high incidence of MRSA infections, patients after a recent hospitalization, dialysis patients, and patients admitted from skilled nursing facilities, based on national recommendations and local epidemiology.

The administration is determined according to the antibiotic half-life. For most frequently used antibiotics, such as cefazolin or cefoxitin, intravenous administration 30–60 min before incision ensures effective tissue concentrations at the time of incision [168]. Redosing during surgery is required when the operative procedure lasts for more than 4 h or there is > 1.5 L blood loss [149, 169]. Vancomycin should be administered within 120 min before the incision, and given over 1 h for a 1 g dose (longer, if the dose is higher). Redosing is generally not required for antibiotics with a long half-life (e.g., fluoroquinolones, metronidazole, vancomycin).

Regarding empiric therapy before causative bacteria and susceptibilities are known, the optimal antibiotic choice should be based on the infection source, expected pathogens, the patient’s clinical condition, local epidemiology, and individual patient risk factors for MDR bacteria. Knowledge of patients’ risk factors for MDR bacteria is essential [1]. Treatment guidelines informed by local epidemiology and resistance patterns should be developed and implemented consistently according to ASP principles. Identifying the correct antibiotic(s) for a particular patient can be complex. Although the susceptibility of bacteria involved in community-acquired infections is usually substantially higher and broader than those involved in HAIs, clinicians often recommend broad-spectrum antibiotics for severe community-acquired infections to avoid “missing anything”. Whereas the spectrum of activity may be appropriate in doing so, the likelihood is over-treatment because narrow-spectrum antibiotics are equally effective in most cases. By contrast, for HAIs, the best course of action is empiric broad-spectrum therapy, including an antifungal agent in some circumstances [170, 171], with later de-escalation to tailored therapy once microbiology data are available [172, 173].

**Fig. 13:**
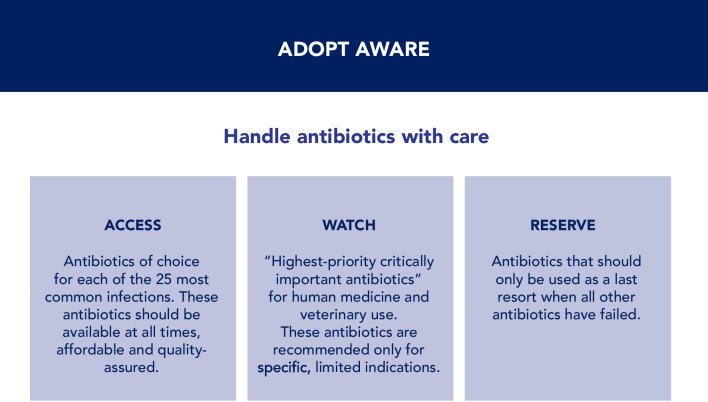
AWaRe classification

In 2017, the WHO Expert Committee on Selection and Use of Essential Medicines established the AWaRe (Access, Watch, Reserve) classification of antibiotics to aid antibiotic stewardship efforts globally at all levels of care. This classification system groups antibiotics into the aforementioned three categories, based on treatment of common bacterial infections and their impact on AMR, focusing on the need for appropriate use. The AWaRe classification [174] was updated in 2021 to include an additional 78 antibiotics that were not previously classified, bringing the total number of classified antibiotics to 258 (Fig. 13).

The “Access” category refers to the antibiotic of choice for each of the 25 most common infections. These antibiotics should be available at all times and places and should be acceptable and affordable. The “Watch” category includes most of the “highest-priority critically important antibiotics”, which are recommended only for specific indications. The “Reserve” category includes antibiotics that should only be used as a last resort (i.e., to treat MDR bacteria) and only when all other antibiotics have failed. WHO recommends reducing the use of antibiotics in the “Watch” and in the “Reserve” groups, and increasing the access and use of antibiotics in the “Access” group, expecting 60% of all antibiotic use in hospitals should come from this group [174]. As a practical matter, this would reduce the global utilization of piperacillin-tazobactam, an antipseudomonal ureidopenicillin that is overused to treat community-acquired infections.

Assessing the severity of infection is a crucial step in evaluating patients for antimicrobial therapy. Early implementation of appropriate empiric antibiotic therapy has a significant favorable impact on outcomes of septic shock, independent of infection site [175]. Whereas critically ill patients benefit from early antibiotic therapy, clinicians managing less severely ill patients may have time to consider carefully and determine the appropriate antibiotic treatment before initiating therapy [175]. Delayed antibiotic prescription, using a wait-and-see approach with reevaluation of the patient, has been reported as a useful tactic to help reduce antibiotic use, particularly for respiratory tract infections [176]. However, some data suggest that hasty antibiotic therapy may be harmful for critically ill surgical patients, where the adequacy of source control is the crucial determinant of survival. A before-after trial [177] compared universal early antibiotic therapy (aggressive approach) to a second period where immediate antibiotic therapy was given only in hypotensive patients. At the same time, other patients underwent therapy only after microbiological confirmation of infection (conservative approach). The aggressive approach was associated with a lower time interval from fever onset and blood culture collection to the start of treatment. The conservative approach was associated with more likely initial appropriate therapy, a shorter duration of therapy, and lower mortality. This differentiation of infection from inflammation can be challenging, especially in critically ill patients, wherein only about one-half of suspected infections are proven. Delaying antibiotics to investigate the cause of sepsis may be of benefit to patients without shock.

Delayed antibiotic prescription is especially useful to help reduce antibiotic use, especially for community-acquired respiratory tract infections [178], many of which have a viral etiology. In a meta-analysis, delayed prescription compared with no antibiotics was associated with similar symptom duration; withholding antibiotics pending pathogen identification and susceptibility testing may be acceptable to reduce unnecessary antibiotic use for viral respiratory tract infections.

By contrast, for patients (both adults and children) with sepsis-related shock and organ dysfunction, delay of appropriate empiric antibiotic therapy can be deleterious [179, 180]; early initiation of antibiotics is crucial for good outcomes. There is a strong correlation between each hour of delay in antibiotic initiation and mortality rates for patients with septic shock [181, 182]. The 2021 Surviving Sepsis Campaign guidelines recommend that adults with possible septic shock or probable sepsis should receive antibiotics as soon as possible, ideally within 1 h from symptom onset [183]. For adults with possible sepsis without shock, a rapid assessment for the likelihood of infectious vs. non-infectious causes of acute illness should be conducted, and antimicrobial agents should be administered within 3 h from the time sepsis was first recognized [183].

The involvement of MDR pathogens in HAIs is a risk factor for inappropriate empiric therapy and, as such, for adverse outcomes. Prior knowledge of colonization caused by MDR bacteria via surveillance cultures improves the likelihood of appropriate initial antibiotic therapy for subsequent HAIs in critically ill patients with blood stream infections or VAP [184, 185]. Additionally, as surveillance cultures have a high negative predictive value for MDR bacteria, early appropriate antibiotic therapy can have antibiotic-sparing potential by reducing use of carbapenems and other antipseudomonal agents compared with a hypothetical guideline-based prescription [185–188]. As such, antibiotic selection pressure on the local ecology may be reduced. A meta-analysis of diagnostic test accuracy revealed that a twice-weekly sampling frequency is the most efficient and that recent surveillance cultures have a higher positive predictive value for bacterial pathogens in VAP [185].

#### Administering antibiotics in adequate doses and appropriate routes

Administering antibiotics in adequate doses should be based on the intrinsic pharmacokinetic (PK) and pharmacodynamic (PD) characteristics of each antibiotic class and the specific agent, and on the specific pathophysiologic characteristics of the patient. Antibiotic PD refers to the relationship between the concentration of the drug and its ability to inhibit bacterial growth. The minimal inhibitory concentration (MIC) is the primary in vitro parameter used to assess the effectiveness of an antibiotic against its target bacteria. In order to obtain a therapeutic effect, the concentration at the site of infection should exceed the MIC against the target bacteria for at least 40% of the dosing interval, and ideally longer (if killing is time-dependent) or by > tenfold (if killing is concentration-dependent) [189]. Antibiotic PK describes how antibiotics are absorbed, distributed, metabolized, and eliminated from the body, which in turn determines the time course and concentration of antibiotics in serum and tissues and at the site of infection. Suboptimal concentrations at the target site may have important clinical consequences such as therapeutic failure and promotion of AMR development, especially when clinical isolates have borderline in vitro susceptibility [190].

Clinical- and antibiotic-related factors can contribute to a differential distribution of antibiotics at the target site [191]. Knowledge of PK/PD of each antibiotic may provide a more appropriate definition of optimal dosing regimens in terms of both dose and administration interval [192]. The concentration gradient between plasma and the site of action may be of high relevance in cases of MDR bacterial infection. For example, data suggest that increased doses of ceftazidime, meropenem, and imipenem-cilastatin are required to reach target attainment in patients with severe intra-abdominal infections [193–195].

Critically ill patients are at high risk of infections, risking life-threatening sepsis and multiple organ dysfunction syndrome. The pathophysiology of sepsis and septic shock can have a major effect on PK parameters. Knowledge of pathophysiologic effects on PK/PD is essential for optimizing antibiotic treatment in critically ill patients with sepsis or septic shock [196, 197]. Hepatic or especially renal dysfunction are conditions where PK changes and dosage reduction may be needed.

The dosing frequency of an antibiotic is determined by the concepts of time-dependent vs. concentration-dependent activity. For example, beta-lactam antibiotics exhibit time-dependent activity, whereby optimal bactericidal activity is achieved when antibiotic concentrations are maintained above the MIC over prolonged periods of time. For this reason, the serum concentration of the antibiotics should exceed the MIC for at least 40% (optimally 70%) of the dosing interval [196]. Higher dosing frequency, prolonged infusions, and continuous infusions achieve this effect and optimize beta-lactam activity [196]. By contrast, antibiotics having concentration-dependent activity are ideally administered to achieve a high peak plasma concentration. For these antibiotics, the peak serum concentration:MIC, not the time above the MIC (fT > MIC), is more closely associated with efficacy [196]. Despite the ideal method of administration and the preferred dosing schemes of aminoglycosides being once-daily dosing for most therapeutic indications, especially in critically ill patients [198], aminoglycoside nephrotoxicity is due to their uptake saturation and a direct vasoconstrictive effect on the renal cortical microcirculation. Thus, limiting aminoglycoside exposure to the renal cortex, by limiting administration to once-daily, reduces the risk of nephrotoxicity [34, 199].

In patients with septic shock, administering a first “loading” dose is probably as fundamental as the timing of administration, depending on the antimicrobial agent [34]. The volume of distribution (*V*_D_) of hydrophilic agents (such as beta-lactams, aminoglycosides, and glycopeptides) in septic shock patients may be increased due to increased microvascular endothelial permeability, expanding the extracellular fluid compartment. Loading doses of beta-lactams, aminoglycosides (especially with once-daily dosing) or glycopeptides are recommended to maximize the therapeutic effect [196].

Once initiated, the antibiotic regimen should be reassessed at least daily, given that fluctuating organ function, common in critically ill patients, may substantially affect antibiotic exposure. For example, lower doses of antibiotics excreted in urine should be administered in the presence of impaired renal function, whereas higher than standard doses should be administered in patients with augmented renal clearance (e.g., burn patients, obesity) [34]. Antibiotic therapy represents a challenge for obese patients because of altered PK/PD [200]. Obesity increases V_D_, especially for lipophilic antibiotics, which can lead to lower-than-expected plasma antibiotic concentrations. Augmented renal clearance is frequent. In addition, fatty infiltration of the liver may impair hepatic function. In general, regardless of body mass, the dosing of lipophilic antibiotics should be based on total body weight, or adjusted body weight for hydrophilic antibiotics. Individualized dosing, supported by laboratory testing, is essential owing to patient heterogeneity and clinical fluctuation.

Recently, a revised consensus guideline and review on therapeutic monitoring of vancomycin for serious MRSA infections was published by the American Society of Health-System Pharmacists, the IDSA, the Pediatric Infectious Diseases Society, and the Society of Infectious Diseases Pharmacists. This consensus revision evaluates the current scientific data and controversies associated with vancomycin dosing and serum concentration monitoring for serious MRSA infections (including but not limited to bacteremia, sepsis, infective endocarditis, pneumonia, osteomyelitis, and meningitis) and provides new recommendations based on recent available evidence [201].

Oral antibiotic administration has been shown to decrease the cost and length of hospitalization [202, 203]. The general guidance for the timing of intravenous-to-oral switching of antibiotics provided the gastrointestinal tract is functional, includes defervescence and clinical improvement with or without improvement in laboratory markers [204]. Numerous antibiotics with high oral bioavailability can be considered for switching, and the switch need not be to the same agent. Many serious infections can now be treated successfully with partial oral antibiotic therapy [205]. However, the switch to oral antibiotics should not lead to an antibiotic therapy which is longer than that used for parenteral therapy. Actually, it is increasingly evident that prescribing oral antibiotics could influence gut microbiome dynamics, promoting more strongly AMR [206].

#### Initiating, as soon as possible, targeted treatment based on the results of culture and susceptibility testing

Microbiologic tests play a crucial role in selecting targeted antibiotic therapy. This testing allows clinicians to tailor the spectrum of the antibiotic, broadening if the initial choice was too narrow, but more commonly narrowing an empiric regimen spectrum that was too broad, known as *de-escalation*. Antibiotic therapy reassessment based on microbiologic culture and susceptibility testing supports ASP and is associated with improved outcomes in severe infections [34].

The de-escalation tactic involves transitioning from a broad-spectrum empiric antibiotic regimen to a narrower-spectrum regimen, or reducing the number of antibiotics used in combination therapy [207], or to monotherapy. The rationale of de-escalation is to avoid broad-spectrum antibiotics whenever possible, diminishing selection pressure and ultimately the prevalence of MDR bacteria, but the practice is controversial as data are scant [208]. The data are strongest for patients with VAP, with higher survival rates reported in several studies [209, 210], obtaining sputum samples before antibiotic administration is a crucial facet to make de-escalation possible. De-escalation has been embraced as part of ASP.

The MIC, the lowest antimicrobial agent concentration that inhibits microbial growth, can be determined by different methods, such as broth or agar dilution, and disk or gradient diffusion. The MIC value, expressed as mcg/mL, is often translated by clinical microbiology laboratories as “susceptible,” “intermediate,” or “resistant” according to defined “breakpoints” established by the Clinical and Laboratory Standards Institute (CLSI, Wayne, PA, USA) or “susceptible,” “susceptible, increased exposure,” or “resistant” according to the criteria of the European Committee on Antimicrobial Susceptibility Testing (EUCAST, Vaxjo, Sweden) [34].

Rapid diagnostics may contribute in furtherance by limiting unnecessary initiation of broad-spectrum therapy, thus decreasing the need for subsequent de-escalation [211, 212]. Most of the commercially available rapid detection methods for MDR bacteria include genotyping that relies on the detection of resistance genes [213] based on DNA sequencing. Genotypic methods may be used in conjunction with phenotyping [214], but genotypic methods in current clinical use should be regarded as supplemental to traditional phenotypic antimicrobial susceptibility testing owing to several limitations. Genotyping can effectively predict AMR, but does not inform susceptibility testing. In addition, the panel of resistance determinants is small, so other resistance determinants may not be detected. Moreover, with genotypic methods there is also the possibility of overestimating AMR, because the presence of a resistance gene is not necessarily associated with the phenotypic expression of resistance (the gene could be inactivated or not expressed).

The greatest advantage of genotyping is undoubtedly speed, with turnaround times of 1–4 h. Employment of comparative genomics, probes, microarrays, nucleic acid amplification techniques, and deoxyribonucleic acid sequencing should allow for the detection of multiple resistance genes or variants simultaneously. However, a logistic challenge in practice is that when new antibiotics are marketed, there may be a lag before methods to measure in vitro susceptibility are validated for clinical use, which may limit the initial clinical use of new agents [215].

Rapid diagnostic testing for possible pathogens is considered indispensable for ASPs. Coupled with prompt, appropriate therapy, antibiotic use is decreased, mortality is reduced, hospital stays are shortened, and cost is lowered [216–218]. The lack of availability of modern diagnostic tests represents an important barrier in low-resource settings [219].

#### Using the shortest duration of antibiotics based on evidence

The duration of antibiotic therapy prescribed in daily practice is often longer than recommended by guidelines [220]. WHO [220] recommends against prolonging the administration of SAP after surgical intervention to prevent SSIs, based on a meta-analysis [220] of 69 randomized controlled trials (RCTs) investigating the optimal duration of SAP. For clean and clean-contaminated procedures, CDC guidelines recommend not to give additional doses of prophylactic antibiotics after the surgical incision has been closed in the operating room, even in the presence of a drain [109]. Updated guidelines of IDSA and SHEA recommend stopping all SAP at incision closure, regardless of procedure type or duration [221].

de Jonge et al*.* examined the effect of continued SAP on the rate of SSI [222]. Eighty-three RCTs were evaluated; 52 (19,273 participants) were included in the primary meta-analysis. No conclusive evidence for the benefit of the post-operative continuation of antibiotic prophylaxis (vs. discontinuation) was identified. When combined with a comprehensive approach to best practices in SSI prevention, post-operative continuation of SAP produced no additional benefit in reducing the incidence of SSI in any surgical setting. In a 2019 multicenter retrospective cohort study [223], increased duration of antibiotic prophylaxis was associated with a higher risk of acute kidney injury and CDI, but no reduction in SSIs.

A study of 34 urban and rural South African hospitals demonstrated that implementation of process improvement initiatives and principles targeted to institutional needs, utilizing pharmacists, effectively improved SAP guideline compliance and sustainable patient outcomes [224]. Efforts to shorten antibiotic therapy duration in hospital practice are a growing area of focus for ASPs [225]. However, Langford et al*.* showed that ASP advice to stop antibiotics or reduce their duration was accepted less often than advice to start or increase antibiotic exposure [226].

Shortening the duration of antibiotic therapy is a crucial tactic for reducing unnecessary inpatient antibiotic use, where antibiotic pressure is intense [227]. Although there are circumstances that may require prolonged antibiotic therapy (e.g., endocarditis, osteomyelitis), the duration of antibiotic therapy should always be as brief as possible. Regarding intra-abdominal infections, the STOP-IT trial [228] demonstrated that, in the setting of adequate source control, 4 days of antibiotic therapy was non-inferior to 8 days of therapy. In the DURAPOP randomized clinical trial [229], critically ill patients with post-operative intra-abdominal infections treated with a short course of antibiotics (8 days) showed similar outcomes compared with those treated for 15 days.

Antibiotic therapy of up to 21 days for VAP and hospital-acquired pneumonia (HAP) was used historically until several prospective studies demonstrated the effectiveness of shorter (7–8 days) therapy with no differences in mortality, intensive care unit (ICU) stay, mechanical ventilation-free days or organ failure-free days [230, 231]. The 2017 European Society of Clinical Microbiology and Infectious Diseases (ESCMID) guidelines and the 2016 IDSA guidelines [232] both recommend 7 days of therapy for HAP/VAP. Ongoing studies [233] are determining if therapy duration could be reduced further.

Bacteremia caused by Enterobacterales has been treated traditionally with 2 weeks of antibiotics. Recent RCTs and meta-analyses investigating shorter (7–8 days) versus longer antibiotic courses (14–15 days) in patients with gram-negative bacteremia (mostly of urinary tract origin) demonstrate non-inferiority [234–239]. Regarding acute uncomplicated cellulitis, evidence also suggests that prolonged courses may be unnecessary and that 5 days of treatment may be sufficient [240]. IDSA guidelines recommend a 5-day antibiotic therapy duration for uncomplicated cellulitis, but may be extended if the infection has not improved within that time frame [241].

Generally, in critically ill patients, decisions about antibiotic therapy duration should be individualized, taking into account patient parameters such as severity of illness, the site and type of infection, whether source control has been achieved, whether PK has been optimized, and clinical response [242]. Procalcitonin (PCT) may be useful to guide antibiotic therapy in the ICU. PCT-guided treatment can reduce the duration of therapy and length of hospital stay in adult critically ill patients with sepsis [243, 244]. Based on apparent benefit and no obvious undesirable effects, the 2021 Surviving Sepsis Campaign guidelines suggest using PCT along with a clinical evaluation to decide when to discontinue antibiotics in adults with an initial diagnosis of sepsis or septic shock and adequate source control, if the optimal duration of therapy is unclear and if PCT is available [183].

#### Achieving source control by identifying and eliminating the source of the infection or reducing the bacterial load

Source control aims to eliminate the source of infection, reduce the bacterial inoculum, and correct anatomic derangements to restore physiologic homeostasis. Additionally, it involves draining abscesses or infected fluid collections, debriding necrotic tissue, or removing contaminated medical devices, all being situations where antibiotics alone have limited efficacy.

Source control is crucial in the management of surgical infections, particularly intra-abdominal and soft tissue infections. Adequate source control achieved by the index operation allows for a shorter course of antibiotic therapy, thereby improving patients’ outcomes, including lower risk of organ dysfunction [245, 246]. In the setting of uncomplicated intra-abdominal infections, such as uncomplicated appendicitis or cholecystitis, post-operative antibiotic therapy is not necessary if source control is adequate [246]. In the setting of complicated intra-abdominal infections, a short course of antibiotic therapy is always suggested even if source control is adequate [228, 229].

In some circumstances, organizational determinants may influence the excessive use of antibiotics. For example, acute cholecystitis should be managed by early cholecystectomy [247]. Nevertheless, because operating room availability is at a premium in many centers, acute cholecystitis cases are sometimes managed by percutaneous drainage or a delayed cholecystectomy, requiring a longer duration of antibiotic therapy.

The urgency (but not the need, ultimately) for source control is determined by the affected organ(s) and the rapidity at which underlying physiological stability deteriorates. Prompt source control may also be important for indolent infections (e.g., infected medical devices). A challenging management problem is central venous catheters associated with catheter-related blood stream infections. In these cases, removal of the catheter (required if the pathogen is *Pseudomonas* spp., *S. aureus* or fungal) constitutes source control. There is little reason to delay source control, even for a few hours, in patients with sepsis [248–250]. The 2021 Surviving Sepsis Campaign guidelines [183] recommend identifying the anatomic source of infection and implementing source control (if amenable) as soon as possible. Delays of as little as 6 h in the setting of sepsis or septic shock have been associated with increased mortality. A multicenter cohort study (2013–2017) of hospitalized adults with community-acquired sepsis (according to SEPSIS-3 definitions) undergoing source control procedures [251] showed that source control within 6 h was associated with a reduced risk of 90-day mortality. In a post hoc analysis of a multicenter observational study (Abdominal Sepsis Study, AbSeS) [252], urgent, successful source control was associated with improved survival, whereas appropriateness of empiric antibiotic treatment was not, suggesting that source control is determinative of outcome for patients with sepsis of abdominal origin. Prompt source control may also be important for other infections [253]. A prospective international cohort study of adult patients (≥ 18 years old) with hospital-acquired blood stream infections treated in ICUs (June 2019–February 2021) reported mortality of 37%. Failure to achieve source control, if required, was associated with death in a multivariable logistic regression model.

Some patients are prone to persistent or recurrent sepsis, despite initial attempts at source control [254]. Index source control procedures may fail up to 25% of the time in abdominal sepsis with shock [255]. Timely surgical re-intervention provides the only option that significantly improves outcomes. Failure of source control may be caused by incomplete initial source control, particularly if contamination is ongoing [256]. Failure of source control can be difficult to diagnose. Therefore, monitoring the success of source control is crucial, with a high index of suspicion if a patient does not improve. Most often, diagnosis is based on a lack of clinical improvement (persistent signs and symptoms of inflammation) and confirmed by imaging.

#### Supporting surveillance of HAIs and AMR, monitoring of antibiotic use, consumption, and the quality of prescribing

Surveillance and prevalence studies to determine the frequency of HAIs are crucial tactics of a strategy to reduce HAIs and contain AMR. Data on HAI prevalence allow hospitals to measure the effectiveness of IPC activities; audits and feedback are used to drive change, improving quality and safety. The European Healthcare-Associated Infections Surveillance Network (HAI-Net) [257], coordinated by the ECDC, provides surveillance of HAIs. The main priorities of HAI-Net are the coordination of European point prevalence surveys of HAIs and antimicrobial use in acute care hospitals and long-term care facilities, surveillance of SSIs, and surveillance of HAIs in ICUs. In the USA, the CDC National Healthcare Safety Network (NHSN) [258] is the most widely used HAI tracking system. NHSN provides facilities and governmental entities with data needed to identify problem areas, measure progress of prevention efforts, and ultimately eliminate HAIs. In addition, NHSN allows tracking of blood transfusion safety errors and important healthcare process measures such as personnel influenza vaccination status, and IPC adherence rates. Surveillance of MDR bacteria provides a basis for taking action to control AMR. Consistent data on the incidence and prevalence of MDR bacteria and geographic patterns related to AMR guide patient treatment and monitor the effectiveness of interventions.

A recent joint publication by ECDC and the WHO Regional Office for Europe reported AMR rates in Europe, using data from invasive bacterial isolates [259]. Carbapenem resistance in *K. pneumoniae* and vancomycin resistance in *E. faecium* increased during 2016–2020. Moreover, high rates of resistance to third-generation cephalosporins and high rates of carbapenem-resistant *Acinetobacter* spp. and *P. aeruginosa* in several European Region countries were identified.

In 2015, WHO launched the Global Antimicrobial Resistance and Use Surveillance System (GLASS), a collaborative effort to standardize AMR surveillance worldwide. Since its launch, GLASS has expanded its coverage and as of 2021, 109 countries and territories worldwide have contributed data to GLASS [260]. High rates of AMR to first-line antibiotics were reported by most countries and in some countries even to last-resort antibiotics. GLASS data demonstrate that globally, carbapenem-resistant bacteria are a serious concern. High rates of carbapenem-resistant *Acinetobacter* spp. and *K. pneumoniae*, resistance to third-generation cephalosporins in Enterobacterales, MDR and XDR tuberculosis, and MRSA will require ongoing close monitoring.

Inappropriate antibiotic use is a main driver of AMR [261]. Data on antibiotic utilization (volume and appropriateness) are essential to evaluate the impact of ASPs. Antibiotic consumption and appropriateness of use can be measured at different levels from nationally down to the prescriber level, allowing informed, focused efforts to reduce unnecessary or inappropriate use [262]. The most common metric to monitor antibiotic consumption is based on the concept of the defined daily dose (DDD). The DDD is the average maintenance dose per day of an antibiotic used in adults for its primary indication. Expressing antibiotic consumption in DDD/1000 patient-days allows comparison regardless of differences in individual antibiotic choices, measuring changes over time to assess the impact of ASP interventions. Between 2000 and 2015, antibiotic consumption expressed in DDD increased by 65% from 21.1 to 34.8 billion DDDs, while the antibiotic consumption rate increased by 39%, from 11.3 to 15.7 DDD/1,000 inhabitants/day) in 76 countries worldwide [263]. Of particular concern was the rapid increase in the use of last-resort compounds, both in high-income countries and LMICs, such as glycylcyclines, oxazolidinones, carbapenems, and polymyxins.

#### Educating staff and improving awareness

One of the goals of the WHO Global Action Plan on Antimicrobial Resistance [49] is to improve awareness and understanding of AMR through effective communication, education, and training. To address AMR, all prescribers must become stewards of antibiotics by prescribing appropriately and educating colleagues and patients on their proper use. The goal of raising awareness is to change behaviors that fuel AMR. Not only can behaviors, beliefs, and practices regarding antibiotic use be inappropriate, there are misconceptions about the concept of AMR itself and its emergence, dissemination, and impact. Clinical leaders should promote awareness by encouraging an institutional culture of patient safety and responsible use where clinicians are persuaded, rather than constrained, to be compliant with antibiotic prescribing measures. Strong patient safety cultures promote education, collaboration, and engagement. Patients must also be engaged with information about the social cost of AMR and the individual benefits of targeted therapy.

The ultimate goal of any stewardship program should be to stimulate a behavioral change in prescribing practices [264]. It is important to incorporate fundamental antimicrobial stewardship, diagnostic stewardship, and IPC principles in under- and post-graduate training and education in order to provide confidence, skills, and expertise in the field of infection management [265]. The education of prescribers is pivotal to convince clinicians to use antibiotics appropriately [1], by respecting correct prescribing practices and following IPC recommendations. There is an urgent call for the integration of antimicrobial stewardship teaching at the undergraduate level of medical education to train future prescribers on this critical aspect of public health. Proper undergraduate education on rational antibiotics use would enable health professional graduates to enter clinical practice with adequate competencies to become rational prescribers [266]. However, although education to intensify AMR prevention is fundamental, without concurrent interventions education alone is of little value. Diagnostic uncertainty, fear of clinical failure or potential litigation, time pressure, or organizational contexts can complicate antibiotic prescribing decisions.

A cross-sectional study of perceptions and practices of physicians and pharmacists regarding antibiotic misuse at primary care centers in the Middle East reported a number of misconceptions and inappropriate practices relating to antibiotic use in Qatar by patients and healthcare providers [267].

Interestingly, the study found that about a third (29.2%) of physicians felt they were often under pressure by patients to prescribe antibiotics. Physicians who are overworked, underinformed, or pressured tend to overprescribe antibiotics and thereby contribute to the spread of AMR. Patients often expect to be prescribed antibiotics, and this pressure can be difficult for physicians to ignore. However, physicians' communication with patients influences their satisfaction more than the actual receipt of antibiotics, especially when patients are asked by their physician to contact them if symptoms do not improve [268]. Therefore, these findings suggest that educating patients about their diagnosis and course of treatment may result in reduced demand for unwarranted antibiotics [267].

#### Supporting multidisciplinary ASPs and enhancing collaboration of healthcare professionals from various disciplines

The promotion of ASPs is pivotal to ensure more standardized and responsible antibiotic use within a healthcare facility [269]. ASPs promulgate and implement best practices to prescribe, administer, monitor, and dispose of antibiotics. However, practices for implementing ASPs may vary based on local culture, policies, and resources. Some hospitals still lack formal ASPs, but even established programs can struggle with sufficient resources and gaining acceptance [270]. The effectiveness of measures to reduce excessive antibiotic prescribing to hospital inpatients and the impact of these measures on reducing the incidence of AMR or CDI have been evaluated [271]. Analyzed measures were able to reduce AMR and HAIs and improve clinical outcomes. Restrictive interventions were recommended when the need to intervene is considered urgent, but over the long term (6 months or more), persuasive measures are equally effective [271].

Promoting ASPs across clinical specialties is crucial to ensuring standardized, rational antibiotic use within a facility as well as a healthcare system [272]. Collaboration allows sharing of knowledge and widespread diffusion of best practices. Timely and accurate reporting of susceptibility test results allows the selection of appropriate targeted therapy and may help reduce broad-spectrum antimicrobial use. ASPs can provide periodic reports on AMR and identify the local microbiological epidemiology for both phenotypic and genotypic analyses [273]. This can impact greatly the choice of empiric therapy. When involved in an ASP, clinical prescribers with knowledge of infectious diseases may help refine antibiotic policy based on local data, audit antibiotic prescribing, provide feedback, integrate best practices of antibiotic use, and act as “champions” among colleagues. Such a champion model has been applied previously to surgical safety implementations in general, such as surgical checklists, and plays a key role in successful quality improvement at the hospital level [274].

Surgeons are responsible for many of the processes that impact the risk for SSIs and play a key role in their prevention. Surgeons are also at the forefront in managing patients with infections, often providing prompt source control and appropriate antibiotic therapy, and are directly responsible for their outcomes. In this context, the direct involvement of surgeons is of utmost importance [270].

Infections are the main factor contributing to ICU mortality. Intensivists have a crucial role in preventing and treating AMR in critically ill patients. Intensivists prescribe antimicrobial agents to challenging patients and thus are at the forefront of successful ASPs [275]. Emergency Departments (EDs) represent a particularly important setting for addressing inappropriate antimicrobial prescribing practices, given the frequent use of antibiotics in this interface between the community and the hospital. Therefore, ED practitioners should also be involved in ASPs [276, 277]. An essential participant in ASPs, often unrecognized and underutilized, is the staff nurse. Nurses are first-responders, crucial communicators, and 24-h guardians of patient status [278]. Their role is becoming formalized in implementing and operating ASPs [278], performing numerous functions that are integral to success. Without adequate support and resources from healthcare administrators, the ASP will not function optimally, in that these programs do not generate revenue. The engagement of healthcare administrators has been confirmed as a key factor for both developing and sustaining an ASP [270].

Successful ASPs can reduce the incidence of infections and colonization with MDR bacteria, including CDI among inpatients [15]. The best means to improve ASP programs is to create a collaborative environment, including all prescribing practitioners [86, 270], to exchange knowledge on best practices and diagnostic capacity.

Due to the challenges posed by the development of new antibiotics, the emergence of MDR bacteria is likely to outpace the introduction of new drugs to combat them. Thus, it may be important to focus on alternative non-antibiotic measures to address AMR [279]. Health information technology is a novel approach to optimize antibiotic use in the healthcare setting, although computerized decision support for hospital antibiotic use may not work in all settings [280]. Curtis et al*.* demonstrated the utility of computerized decision support of antibiotic usage and even in reduction of mortality in hospital settings [281].

Whereas clinical research should work toward developing new management techniques and therapies to address AMR, physicians should continue to preserve the use of antibiotics as much as possible. Additionally, public health campaigns aimed at promoting awareness about the responsible use of antibiotics and IPC measures can also be crucial in reducing the spread of MDR bacteria.

**Fig. 14:**
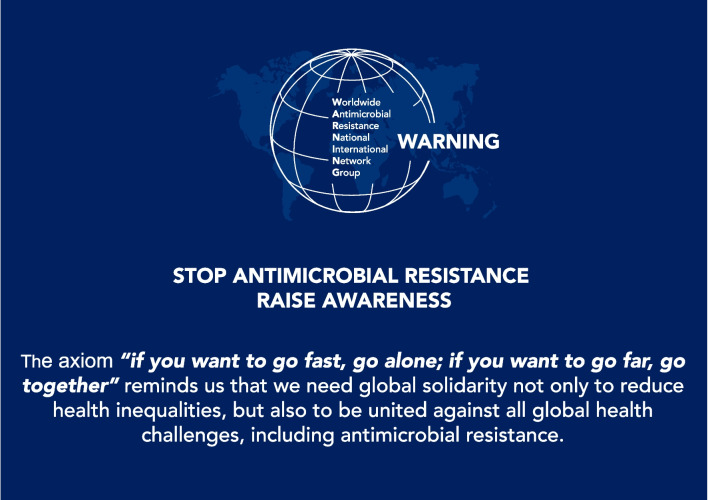
Supporting a cohesive and multidisciplinary approach

This document confirms the mission of the Global Alliance for Infections in Surgery, promoting standards of care in managing surgical infections through a cohesive and multidisciplinary approach. The axiom “if you want to go fast, go alone; if you want to go far, go together” reminds us that we need global solidarity not only to reduce health inequalities, but also to be united against all global health challenges, including AMR (Fig. 14).

## Conclusions

Appropriate use of antibiotics should be integral to good clinical practice and standards of care. Inappropriate antibiotic use as well as poor IPC practices are contributing to the development and spread of AMR. Antibiotics should be treated as a global public good on the verge of scarcity; there is a global collective responsibility to preserve them in order to avoid countless future victims of MDR infections. Infections, especially those with MDR bacteria, compromise the success of all medical practitioners, including surgeons. A technically proficient surgery will be unsuccessful if the patient succumbs to a HAI that cannot be treated. Through collaborative initiatives and a united front, a future of effective antimicrobial therapy can be envisioned for generations to come.

## Data Availability

Not applicable.

## Abbreviations

AMR: Antimicrobial resistance
ASPs: Antimicrobial Stewardship Programs
CDC: Centers for Disease Control and Prevention
CDI: *Clostridioides difficile* infection
COVID-19: Coronavirus disease 2019
CRAB: Carbapenem-resistant *Acinetobacter baumannii*
CRE: Carbapenem-resistant enterobacterales
CRPA: Carbapenem-resistant *Pseudomonas aeruginosa*
DDD: Defined daily doses
ECDC: European Centre for Disease Prevention and Control
EDs: Emergency departments
GLASS: Global antimicrobial resistance and use surveillance system
HAIs: Hospital-acquired infections
HAP: Hospital-acquired pneumonia
HCWs: Healthcare workers
Hib: *Haemophilus influenzae* type B
ICU: Intensive care unit
IDSA: Infectious Diseases Society of America
IPC: Infection prevention and control
LMICs: Low- and middle-income countries
MDR: Multidrug-resistant
MIC: Minimal inhibitory concentration
MRSA: Methicillin-resistant *Staphylococcus. aureus*
NAPs: National action plans
PCT: Procalcitonin
PD: Pharmacodynamics
PDR: Pan-drug-resistant
PK: Pharmacokinetics
RCTs: Randomized controlled trials
SAP: Surgical antibiotic prophylaxis
SARS-CoV-2: Severe acute respiratory syndrome coronavirus-2
SHEA: Society for Healthcare Epidemiology of America
SSIs: Surgical site infections
VAP: Ventilator-associated pneumonia
WHO: World Health Organization
XDR: Extensively drug-resistant
